# Supplementary material for: Large deep-sea zooplankton biomass mirrors primary production in the global ocean
Source: Nat Commun. 2020 Nov 27;11:6048. doi: 10.1038/s41467-020-19875-7 (PMC7695708; doi:10.1038/s41467-020-19875-7)
Supplement: Supplementary file 1 — Supplementary Information [file 41467_2020_19875_MOESM1_ESM.pdf]

Supplementary Information

Supplementary Table 1. Regression data of the zooplankton biomass (ZB, mgC·m^{-3}) decrease with depth (z, km) for the different biomes (LnZB= az+b). Northern and southern polar biomes were considered separately because of the large biomasses reported in the north. Mediterranean data are also considered apart because of the oligotrophy of this waters within the Westerlies.

**Biome**	**Equation**	r^2	**p<**	**n**
Trades	LnZB= 0.470-1.334·km	0.646	0.001	476
Westerlies	LnZB= 0.930-1.023·km	0.440	0.001	924
Polar North	LnZB= 2.436-0.878·km	0.159	0.001	78
Polar South	LnZB= 0.935-1.144·km	0.267	0.001	210
Coastal	LnZB= 0.597-1.131·km	0.622	0.001	262
Mediterranean	LnZB= -0.436-1.354·km	0.649	0.001	596
**All data**	LnZB= 0.568-1.214·km	0.477	0.001	2546

Supplementary Table 2. Regression data of the zooplankton $\delta^{15}N$ (‰) decrease with depth (m) for the Coastal and Trades biomes.

**Biome**	**Equation**	r^2	**p<**	**n**
Coastal	$\delta^{15}N = 9.897 - 0.243$ depth (km)	0.004	0.666	46
Trades	$\delta^{15}N = 6.006 + 4.230$ depth (km)	0.269	0.001	123

Supplementary Table 3. Respiration rates in the bathypelagic zone obtained during the Malaspina cruise for different size classes using the electron transfer system (ETS) activity as a proxy. "Prot" stands for protein, "dw" for dry weight, "SD" for standard deviation, and "n" the number of samples.

**Station**	**Depth** (m)	**Size fraction** (μm)	**Temperature** (°C)	**sp ETS in situ** ($\mu lO_2 \cdot mgprot^{-1} \cdot h^{-1}$)	**Respiration** ($\mu lO_2 \cdot mgdw^{-1} \cdot h^{-1}$)	**Respiration** (d^{-1})
12	1000-2000	>1000	4.31	3.30	0.66	0.021
12	1000-2000	1000-500	4.31	1.42	0.29	0.009
12	1000-2000	500-300	4.31	0.35	0.07	0.002
35	1000-2000	>1000	2.99	0.79	0.16	0.005
35	1000-2000	1000-500	2.99	2.66	0.53	0.017
35	1000-2000	500-300	2.99	1.96	0.39	0.012
39	1000-2000	>1000	3.10	1.52	0.31	0.010
39	1000-2000	1000-500	3.10	3.85	0.77	0.024
39	1000-2000	500-300	3.10	0.02	0.00	0.000
45	1000-2000	>1000	4.59	0.74	0.15	0.005
45	1000-2000	1000-500	4.59	0.47	0.09	0.003
45	1000-2000	500-300	4.59	1.14	0.23	0.007
60	1000-2000	>300	3.29	3.79	0.76	0.024
69	1000-2000	>1000	2.91	0.27	0.05	0.002
69	1000-2000	>1000	2.91	0.27	0.05	0.002
69	1000-2000	1000-500	2.91	9.83	1.97	0.062
69	1000-2000	500-300	2.91	0.20	0.04	0.001
71	1000-2000	>1000	3.01	3.28	0.66	0.021
71	1000-2000	>1000	3.01	2.39	0.48	0.015
71	1000-2000	>1000	3.01	0.81	0.16	0.005
71	1000-2000	1000-500	3.01	1.69	0.34	0.011
84	1000-1500	>1000	2.47	0.10	0.02	0.001
84	1000-1500	1000-500	2.47	0.07	0.01	0.000
84	1000-1500	500-300	2.47	1.62	0.33	0.010
84	1000-1500	>1000	3.27	0.44	0.09	0.003
84	1000-1500	1000-500	3.27	0.56	0.11	0.004
84	1000-1500	500-300	3.27	6.02	1.21	0.038
92	1000-2000	>1000	3.23	1.27	0.26	0.008
92	1000-2000	1000-500	3.23	0.51	0.10	0.003
92	1000-2000	500-300	3.23	0.92	0.18	0.006
136	1000-2000	>1000	3.29	1.22	0.24	0.008
136	1000-2000	500-1000	3.29	0.40	0.08	0.003
136	1000-2000	500-300	3.29	0.56	0.11	0.004
Average					0.33	0.010
SD					0.37	0.012
n						33

Supplementary Table 4. Values for the different domains and provinces used in this review for the 1000-2000, 2000-3000 and 3000-4000 m layers. N is the number of profiles obtained for each oceanic province and SD is standard deviation.

Domain	Ocean	Province	Biomass at 1000-2000 m mgC·m^{-2}	Remineralization 1000-2000 m gC·m^{-2}·y^{-1}	SD	n	Area of provinces 10^6 Km^2 Longhurst (1995)	Remineralization 1000-2000 m TgC·y^{-1} This study	Primary Production gC·m^{-2}·y^{-1} Longhurst (1995)
Trades	Atlantic	NATR	32.6	0.12	0.41	3	8.27	0.98	106
		WTRA	26.8	0.1	0.04	3	5.36	0.52	130
		SATL	36.5	0.13	1.13	11	17.77	2.37	75
Trades	Indian	MONS	199.5	0.73	0.37	30	14.21	10.35	105
		ISSG	78.0	0.28	0.22	15	19.25	5.48	71
Trades	Pacific	NPTG	67.2	0.25	0.29	5	21.09	5.17	59
		PNEC	120.0	0.44	0.23	5	8.17	3.58	107
		PEQD	51.5	0.19	0.12	4	10.34	1.95	113
		WARM	57.6	0.21	0.02	3	16.78	3.53	82
		ARCH	288.0	1.05		1	8.84	9.29	100
Westerlies	Atlantic	NADR	402.9	1.47	2	12	3.5	5.15	240
		GFST	2784.0	10.16		1	1.1	11.18	178
		MEDI	47.3	0.17	0.53	29	3.08	0.53	216
		NASE	81.7	0.3	0.86	8	4.45	1.33	122
Westerlies	Pacific	PSAW	2280.0	8.32	2.2	14	2.9	24.13	264
		KURO	1955.0	7.14	3.1	2	3.7	26.4	193
		NPSW	139.4	0.51	0.71	8	3.93	2	109
		SPSG	50.4	0.18	0.29	7	37.29	6.86	87
Westerlies	Southern	SSTC	241.8	0.88	0.49	17	16.84	14.86	136
Westerlies	Southern	SANT	241.8	0.88	0.59	27	30.25	26.7	120
Coastal	Indian	REDS	9.6	0.04	0.02	7	0.56	0.02	617
		ARAB	375.1	1.38	0.33	13	2.93	4.06	454
		EAFR	287.7	1.05	0.33	2	3.72	3.91	190
		AUSW	289.8	1.06	0.99	2	2.94	3.11	199
Coastal	Atlantic	BENG	196.7	0.72	0.06	2	1.13	0.81	323
Coastal	Pacific	CCAL	205.2	0.75	0.34	3	0.96	0.72	388
		AUSE	613.1	2.24		1	1.14	2.55	232
Polar	Atlantic	SARC	2593.5	9.47	9.97	2	2.33	22.06	302
		ARCTIC	2051.7	7.49	22.92	8	2.1	15.73	484
Polar	Southern	ANTA	294.5	1.07	0.58	26	8.87	9.53	165
Polar	Southern	APLR	120.9	0.44	0.04	3	1.93	0.85	398
**1000-2000 m**				1.91		274	265.7 (81%)	**225.7**	

(Continued)

Domain	Ocean	Province	Biomass at 2000-3000 m mgC·m^{-2}	Remineralization 2000-3000 m gC·m^{-2}·y^{-1}	SD	n	Area of provinces 10^6 Km^2 Longhurst (1995)	Remineralization 2000-3000 m TgC·y^{-1} This study	Primary Production gC·m^{-2}·y^{-1} Longhurst (1995)
Trades	Atlantic	NATR	17.70	0.06	0.09	2	8.27	0.53	106
		WTRA	10.60	0.04	0.04	2	5.36	0.21	130
		SATL	80.00	0.29	0.37	2	17.77	5.19	75
Trades	Indian	MONS	78.00	0.28	0.11	22	14.21	4.05	105
		ISSG	117.00	0.43	0.2	5	19.25	8.22	71
Trades	Pacific	NPTG	26.50	0.1	0.09	4	21.09	2.04	59
		PNEC	34.10	0.12	0.03	3	8.17	1.02	107
		PEQD	12.00	0.04	0.01	2	10.34	0.45	113
		WARM	27.60	0.1		1	16.78	1.69	82
Westerlies	Atlantic	NADR	102.50	0.37	0.21	8	3.5	1.31	240
		GFST	2556.00	9.33		1	1.1	10.26	178
		MEDI	18.50	0.07	0.03	18	3.08	0.21	216
		NASE	39.00	0.14	0.16	5	4.45	0.63	122
Westerlies	Pacific	PSAW	1168.80	4.27	1.54	9	2.9	12.37	264
		KURO	385.00	1.41		1	3.7	5.2	193
		NPSW	8.80	0.03	0.07	3	3.93	0.13	109
		SPSG	10.20	0.04	0.02	3	37.29	1.39	87
Coastal	Indian	REDS	9.60	0.04	0.04	3	0.56	0.02	617
		ARAB	135.20	0.49	0.1	9	2.93	1.45	454
		EAFR	224.20	0.82		1	3.72	3.04	190
Coastal	Atlantic	BENG	110.50	0.4		1	1.13	0.46	323
Coastal	Pacific	CHIL	115.80	0.42	0.02	2	0.96	0.41	388
Polar		ARCTIC	323.80	1.18	9.26	6	2.1	2.48	484
**2000-3000 m**				0.89		113	192.59 (59%)	**62.74**	

(Continued)

Domain	Ocean	Province	Biomass at 3000-4000 m mgC·m^{-2}	Remineralization 3000-4000 m gC·m^{-2}·y^{-1}	SD	n	Area of provinces 10^6 Km^2 Longhurst (1995)	Remineralization 3000-4000 m TgC·y^{-1} This study	Primary Production gC·m^{-2}·y^{-1} Longhurst (1995)
Trades	Atlantic	NATR	0.62	0.002		1	8.27	0.02	106
Trades	Indian	MONS	46.7	0.17	0.14	10	14.21	2.42	105
		ISSG	78	0.28	0.2	2	19.25	5.48	71
Trades	Pacific	NPTG	26.4	0.1		1	21.09	2.03	59
		PNEC	34.8	0.13		1	8.17	1.04	107
		WARM	7.7	0.03		1	16.78	0.47	82
Westerlies	Atlantic	NADR	67.3	0.25	0.14	9	3.5	0.86	240
		MEDI	9	0.03	0.69	14	3.08	0.1	216
		NASE	19.4	0.07	0.04	4	4.45	0.32	122
Westerlies	Pacific	PSAW	169.2	0.62	0.21	9	2.9	1.79	264
		KURO	29	0.11		1	3.7	0.39	193
		NPSW	19	0.07	0.03	4	3.93	0.27	109
		ARAB	67.1	0.24	0.07	8	2.93	0.72	454
Polar		ARCTIC	51.6	0.19		1	2.1	0.4	484
**3000-4000 m**				0.16		66	114.36 (35%)	**16.31**	

Supplementary Table 5. Domain, ocean and data source information used for the zooplankton vertical distribution review. "This study" refers to data obtained during the Malaspina Expedition.

Domain	Ocean	Province	Authors
Trades	Atlantic	NATR	This study
			Angel and Baker (1982)[1*]
		WTRA	This study
		SATL	This study
			Foxton (1956)[2]
Trades	Indian	MONS	Vinogradov (1970)[3]
			Grice and Hulsemann (1967)[4*]
			Foxton (1956)[2]
			Koppelmann et al. (2000)[5]
		ISSG	This study
			Vinogradov (1970)[3]
			Grice and Hulsemann (1967)[4*]
			Foxton (1956)[2]
Trades	Pacific	NPTG	This study
			Vinogradov (1970)[3]
		PNEC	This study
			Vinogradov (1970)[3]
		PEQD	This study
			Vinogradov (1970)[3]
		WARM	Vinogradov (1970)[3]
		ARCH	Vinogradov (1970)[3]
Westerlies	Atlantic	NADR	Koppelmann and Weikert (1992)[6]
			Koppelmann and Weikert (1999)[7]
			Angel and Baker (1982)[1*]
		GFST	Vinogradov et al (1998)[8]
		MEDI	Weikert and Trinkaus (1990)[9]
			Weikert et al. (2001)[10]
			Koppelmann and Weikert (2007)[11]
			Koppelmann et al. (2004)[12]
			Minutoli & Guglielmo (2012)[13]
		NASE	This study
			Roe (1988)[14*]
			Grice and Hulsemann (1965)[15*]
			Angel and Baker (1982)[1*]
Westerlies	Pacific	PSAW	Vinogradov (1970)[3]
			Yamaguchi et al (2004)[16]
		KURO	Vinogradov (1970)[3]
			Yamaguchi et al (2004)[16]
		NPSW	Vinogradov (1970)[3]
			Yamaguchi et al (2004)[16]
		SPSG	This study
			Vinogradov (1970)[3]
Westerlies	Southern	SSTC	This study
			Foxton (1956)[2]
Westerlies	Southern	SANT	Foxton (1956)[2]
Coastal	Indian	REDS	Wishner (1980)[17*]
			Weikert (1982)[18]
		ARAB	Koppelmann et al. (2000)[5]
			Grice and Hulsemann (1967)[4*]
			Böttger-Schnack (1996)[19]
		BENG	Koppelmann (unpubl.)
		EAFR	This study
		AUSW	This study
Coastal	Pacific	CCAL	Wishner (1980)[17*]
		AUSE	This study
Polar	Atlantic	SARC	Vinogradov et al (1998)[8]
			Grice and Hulsemann (1965)[15*]
		ARCTIC	Richter (1994)[20*]
Polar	Southern	ANTA	Foxton (1956)[2]
Polar	Southern	APLR	Foxton (1956)[2]

Supplementary Table 6. Reviewed values of carbon export and sequestration of particulate organic carbon (POC) flux, active flux due to zooplankton and micronekton, and estimated dissolved organic carbon (DOC) flux used to build Figure 4. Numbers in blue were used as lower and higher values in the range given in Figure 4. Values in red were considered outliers and they were not accounted in the Figure.

System	Flux or Respiration	Depth (m)	Authors	µmolC·m^{-3}·d^{-1}	mmolC·m^{-2}·d^{-1}	mgC·m^{-2}·d^{-1}	gC·m^{-2}·y^{-1}	Comments
**Oligotrophic**	**Mesopelagic**	150	Martin et al. (1987)[21]			35.5	13.0	VERTEX 14-35°N
	**POC flux**	150	Knauer et al. (1990)[22]			31.2	11.4	VERTEX 33°N
		150	Karl et al. (1996)[23]			36.0	13.1	Aver. HOT 1989 (Table 3)
		150	Karl et al. (1996)[23]			35.0	12.8	Aver. HOT 1990 (Table 3)
		150	Karl et al. (1996)[23]			27.0	9.9	Aver. HOT 1991 (Table 3)
		150	Karl et al. (1996)[23]			22.0	8.0	Aver. HOT 1992 (Table 3)
		150	Karl et al. (1996)[23]			25.0	9.1	Aver. HOT 1993 (Table 3)
		150	Hernández-León et al. (2019)[24]			11.3	4.1	Aver. sts 2-7 and 14 (Table 1)
		200	Miquel et al. (2011)[25]				2.4	Aver. 1988-2005 (Table 2)
		150	Buesseler et al. (2007)[26]			18.0	6.6	Hawaii Aloha st.
		150	Buesseler et al. (2007)[26]			18.0	6.6	Hawaii Aloha st.
	**Bathypelagic**	1000	Miquel et al. (2011)[25]				1.6	Mediterranean Aver. 1988-2005 (Table 2)
	**POC flux**	2000	Guidi et al. (2015)[27]				1.44	Range 0.1-9.3
		2000	Henson et al. (2012)[28]				1.74	Range 0-35
		2000	Henson et al. (2012)[28]				0.5	About 0.5 for oligotrophic gyres
		2000	Martin et al. (1987)[21]				0.8	Range 0.8-7.0
		2000	Lutz et al. (2007)[29]				0.2	Range 0.2-7.0
		2000	Honjo et al. (2008)[30]				0.3	Range 0.3-7.3
	**Mesopelagic**	200	Hidaka et al. (2001)[31]			27.9	10.2	Western equatorial North Pacific (3°N)
	**Active flux**	200	Hidaka et al. (2001)[31]			12.2	4.5	Western equatorial North Pacific (3° 30´N)
		200	Ariza et al. (2015)[32]			6.4	2.3	Canary Current
		200	Hernández-León et al. (2019)[24]			3.2	1.2	St 3 South Equatorial Counter Current
		200	Hernández-León et al. (2019)[24]			12.6	4.6	St 6 South Equatorial Current
		200	Hernández-León et al. (2019)[24]			23.1	8.4	St 7 North Equatorial Counter Current
		200	Hernández-León et al. (2019)[24]			12.2	4.5	St 12 Canary Current
	**Mesopelagic**	100-1000	Nagata et al. (2000)[33]		2.9	34.8	12.7	Geomean Table 10.4 Arístegui et al. (2005)
	**respiration**	200-1000	Santana-Falcón et al. (2017)[34]				7.0	Tropical and temperate North Atlantic Ocean
		200-1000	Arístegui et al. (2003a)[35]				35.0	
		>200	Arístegui et al. (2003b)[36]		4	48.0	17.5	Lower range in the Eastern Subtropical North Atlantic gyre
		>200	Arístegui et al. (2003b)[36]		8	96.0	35.0	Higher range in the Eastern Subtropical North Atlantic gyre
		200-1000	Jenkins and Wallace (1992)[37]				39.6	Atlantic Ocean (Sagasso Sea)
		200-1000	Packard et al. (1988)[38]				18.0	Atlantic Ocean (Sagasso Sea)
		200-1000	Arístegui et al. (2003a)[35]				28.8	Atlantic Ocean (Canary Islands)
		200-800	Savenkoff et al. (1993)[39]				26.4	Mediterranean Sea (west)
		200-1000	Lefevre et al. (1996)[40]				14.4	Mediterranean Sea (west)
		200-1000	Baltar et al. (2009)[41]	5.90	4.72	56.6	20.7	North Equatorial Counter Current (ETS in 900-1000 m depth)
		200-1000	Baltar et al. (2009)[41]	2.49	1.992	23.9	8.7	Subtropical Gyre (ETS in 900-1000 m depth)
		200-1000	Baltar et al. (2009)[41]	16.30	13.04	156.5	57.1	North Equatorial Counter Current (ETS in 250-500 m depth OMZ)
		200-1000	Baltar et al. (2009)[41]	13.50	10.8	129.6	47.3	Subtropical Gyre (ETS in 250-500 m depth OMZ)
		150-700	Fernández-Castro et al. (2016)[42]				30.7	Mean value Time series station ESTOC Canary Current
	**Bathypelagic**	>1000	Nagata et al. (2000)[33]		0.1	1.2	0.4	N Subtropical Pacific
	**respiration**	>1000	Packard et al. (1988)[38]				8.0	Sargasso Sea
	**Zooplankton**	150-1000	Hernández-León and Ikeda (2005)[43]		0.21	2.5	0.9	Lower range of values for the mesopelagic zone
	**mesopelagic**	150-1000	Hernández-León and Ikeda (2005)[43]		14	168.0	61.3	Higher range of values for the mesopelagic zone
	**respiration**	150-1000	Hernández-León and Ikeda (2005)[43]		1.7	20.4	7.4	Average value for the mesopelagic zone
		200-900	Hernández-León (unpublished)			5.22	1.9	Coca I cruise. Respiration by day. Oceanic zone Canary Current
		200-900	Hernández-León (unpublished)			12.17	4.4	Coca I cruise. Respiration by day. Oceanic zone Canary Current
		200-900	Hernández-León (unpublished)			10.54	3.8	Coca I cruise. Respiration by day. Oceanic zone Canary Current
		200-900	Hernández-León (unpublished)			9.40	3.4	Coca I cruise. Respiration by day. Oceanic zone Canary Current
		200-900	Hernández-León (unpublished)			10.57	3.9	Coca I cruise. Respiration by day. Oceanic zone Canary Current
		200-900	Hernández-León (unpublished)			26.25	9.6	Coca I cruise. Respiration by day. Oceanic zone Canary Current
		200-900	Hernández-León (unpublished)			18.66	6.8	Coca I cruise. Respiration by day. Oceanic zone Canary Current
		200-900	Hernández-León (unpublished)			10.55	3.9	Coca I cruise. Respiration by night. Oceanic zone Canary Current
		200-900	Hernández-León (unpublished)			20.06	7.3	Coca I cruise. Respiration by night. Oceanic zone Canary Current
		200-900	Hernández-León (unpublished)			4.17	1.5	Coca I cruise. Respiration by night. Oceanic zone Canary Current
		200-900	Hernández-León (unpublished)			12.88	4.7	Coca I cruise. Respiration by night. Oceanic zone Canary Current
		200-900	Hernández-León (unpublished)			8.59	3.1	Coca I cruise. Respiration by night. Oceanic zone Canary Current
		200-900	Hernández-León (unpublished)			26.99	9.9	Coca I cruise. Respiration by night. Oceanic zone Canary Current
		200-900	Hernández-León (unpublished)			21.20	7.7	Coca I cruise. Respiration by night. Oceanic zone Canary Current
	**Zooplankton**	1000-4000	Childress and Thuesen (1992)[44]			0.1	0.05	Hypothetical central oceanic region
	**Bathypelagic**	1000-2250	Koppelmann and Weikert (1999)[7]		0.11	1.3	0.5	Spring
	**Respiration**	1000-2250	Koppelmann and Weikert (1999)[7]		0.32	3.8	1.4	Summer
		>2250	Koppelmann and Weikert (1999)[7]		0.03	0.4	0.1	Spring
		>2250	Koppelmann and Weikert (1999)[7]		0.03	0.4	0.1	Summer
		1000-4000	Hernández-León and Ikeda (2005)[43]		0.32	3.8	1.4	Average value for the bathypelagic zone
		1000-2000	This study				1.9	
		2000-3000	This study				0.9	
		3000-4000	This study				0.2	

Supplementary Table 6 (cont.).

System	Flux or Respiration	Depth (m)	Authors	$\mu molC \cdot m^{-3} \cdot d^{-1}$	$mmolC \cdot m^{-2} \cdot d^{-1}$	$mgC \cdot m^{-2} \cdot d^{-1}$	$gC \cdot m^{-2} \cdot y^{-1}$	Comments
**Productive**	**Mesopelagic**		Karl et al. (1996)[23]			42.0	15.3	Coastal zone (Table 4)
	**POC flux**		Karl et al. (1996)[23]			85.0	31.0	Upwelling zone (Table 4)
		150	Hernández-León et al. (2019)[24]			26.0	9.5	Aver. sts 8 and 11 (Table 1)
		150	Buesseler et al. (2007)[26]			62	22.6	Northwest Pacific Subarctic gyre St K2
		150	Buesseler et al. (2007)[26]			23	8.4	Northwest Pacific Subarctic gyre St K2
	**Bathypelagic**	1000	Helmke et al. (2005)[45]			20.6	7.5	Aver. 4 years NW Africa
	**POC flux**	2000	Henson et al. (2012)[28]				3.0	3-4 for equatorial and Arabian upwelling
		2000	Martin et al. (1987)[21]				7.0	Range 0.8-7.0
		2000	Lutz et al. (2007)[29]				7.0	Range 0.2-7.0
		2000	Honjo et al. (2008)[30]				7.3	Range 0.3-7.3
	**Mesopelagic**	200	Hernández-León et al. (2019)[24]			43.0	15.7	St 9 Guinea Dome
	**Active flux**	200	Hernández-León et al. (2019)[24]			85.0	31.0	St 11 Oceanic upwelling off C. Blanc
	**Mesopelagic**	100-1000	Nagata et al. (2000)[33]		42	504.0	184.0	Santa Monica Basin
	**respiration**	>200	Arístegui et al. (2003b)[36]		5	60.0	21.9	Lower range in the Coastal Transition Zone Canary Current
		>200	Arístegui et al. (2003b)[36]		10	120.0	43.8	Higher range in the Coastal Transition Zone Canary Current
		150-1000	Packard et al. (2015)[46]		3.49	41.9	15.3	Upwelling zone off Perú (Tabla 5)
		150-1000	Packard et al. (2015)[46]		344.93	4139.2	1510.8	Upwelling zone off Perú (Tabla 5)
		150-1000	Packard et al. (2015)[46]		11.46	137.5	50.2	Upwelling zone off Perú (Tabla 5)
		150-1000	Packard et al. (2015)[46]		2.43	29.2	10.6	Upwelling zone off Perú (Tabla 5)
		150-1000	Packard et al. (2015)[46]		2.72	32.6	11.9	Upwelling zone off Perú (Tabla 5)
		150-1000	Packard et al. (2015)[46]		19.57	234.8	85.7	Upwelling zone off Perú (Tabla 5)
		150-1000	Packard et al. (2015)[46]		3.53	42.4	15.5	Upwelling zone off Perú (Tabla 5)
		150-1000	Packard et al. (2015)[46]		6.3	75.6	27.6	Upwelling zone off Perú (Tabla 5)
	**Bathypelagic**	>1000	Packard et al. (1988)[38]				31.3	Pacific Ocean (Costa Rica Dome)
	**respiration**	>1000	Turley and Mackie (1994)[47]		0.4	4.8	1.8	NE Atlantic
		>1000	Nagata et al. (2000)[33]		0.5	6.0	2.2	Subarctic Pacific
		>1000	Packard et al. (2015)[46]		3.07	36.8	13.4	Upwelling zone off Perú (Tabla 5)
		>1000	Packard et al. (2015)[46]		0.38	4.6	1.7	Upwelling zone off Perú (Tabla 5)
		>1000	Packard et al. (2015)[46]		0.34	4.1	1.5	Upwelling zone off Perú (Tabla 5)
		>1000	Packard et al. (2015)[46]		22.51	270.1	98.6	Upwelling zone off Perú (Tabla 5)
		>1000	Packard et al. (2015)[46]		0.79	9.5	3.5	Upwelling zone off Perú (Tabla 5)
		>1000	Packard et al. (2015)[46]		1.28	15.4	5.6	Upwelling zone off Perú (Tabla 5)
	**Zooplankton**	200-900	Hernández-León (unpublished)			22.6	8.3	Coca I cruise. Respiration by day. Upwelling zone Canary Current
	**mesopelagic**	200-900	Hernández-León (unpublished)			90.4	33.0	Coca I cruise. Respiration by day. Oceanic zone Canary Current
	**respiration**	200-900	Hernández-León (unpublished)			21.6	7.9	Coca I cruise. Respiration by night. Oceanic zone Canary Current
		200-900	Hernández-León (unpublished)			62.2	22.7	Coca I cruise. Respiration by night. Oceanic zone Canary Current
	**Zooplankton**	1000-2000	This study				1.0	Coastal
	**bathypelagic**	1000-2000	This study				3.0	Westerlies
	**respiration**							

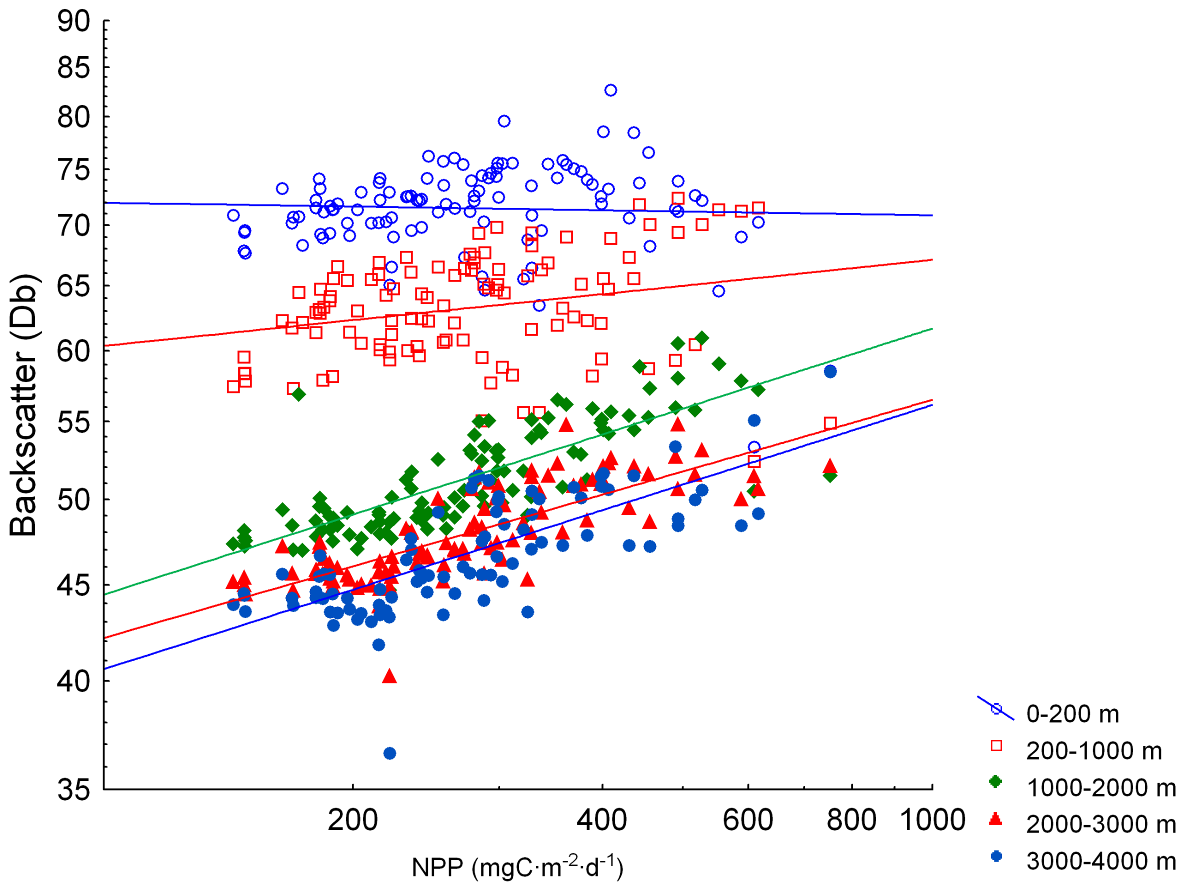

Supplementary Figure 1. Relationships between net primary production and the backscatter signal (dB) obtained from the Lowered Acoustic Doppler Current Profiler (LADCP) attached to the CTD rosette in epipelagic (0-200 m), mesopelagic (200-1000 m), and the upper (1000-2000 m), intermediate (2000-3000 m), and lower (3000-4000 m) bathypelagic layers. A large variability was found in the epipelagic layer (0-200 m depth) in relation to primary production because of the mismatch of measuring biomass or backscatter at a given time during the cruise and the remote sensing average used for primary production in a rather highly dynamic system as the epipelagic zone. Here, the higher primary production was observed in the coastal domain (mainly close to upwelling zones), but this high values were not coupled to high backscatter in the epipelagic zone or even in the mesopelagic zone. However, it was better correlated in the bathypelagic zone as the biomass proxy there is the result of long-term primary production in the upper layers.

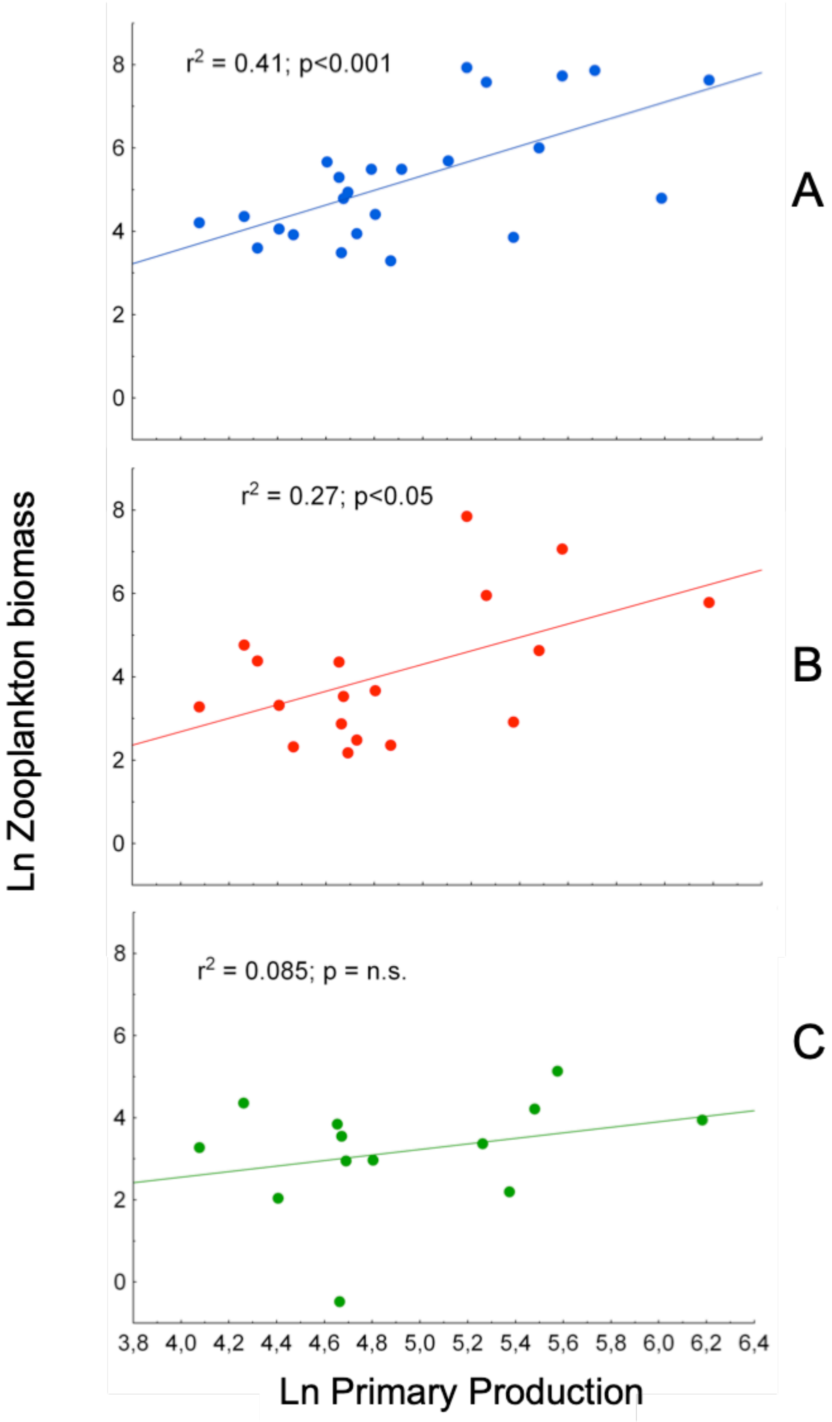

Supplementary Figure 2. Relationships between Longhurst primary production and average zooplankton biomass for the different provinces of Trades, Westerlies, and Polar domains, and excluding the coastal one, for (A) the upper (1000-2000 m), (B) intermediate (2000-3000 m), and (C) lower (3000-4000 m) bathypelagic layers. n.s. stand for not significant.

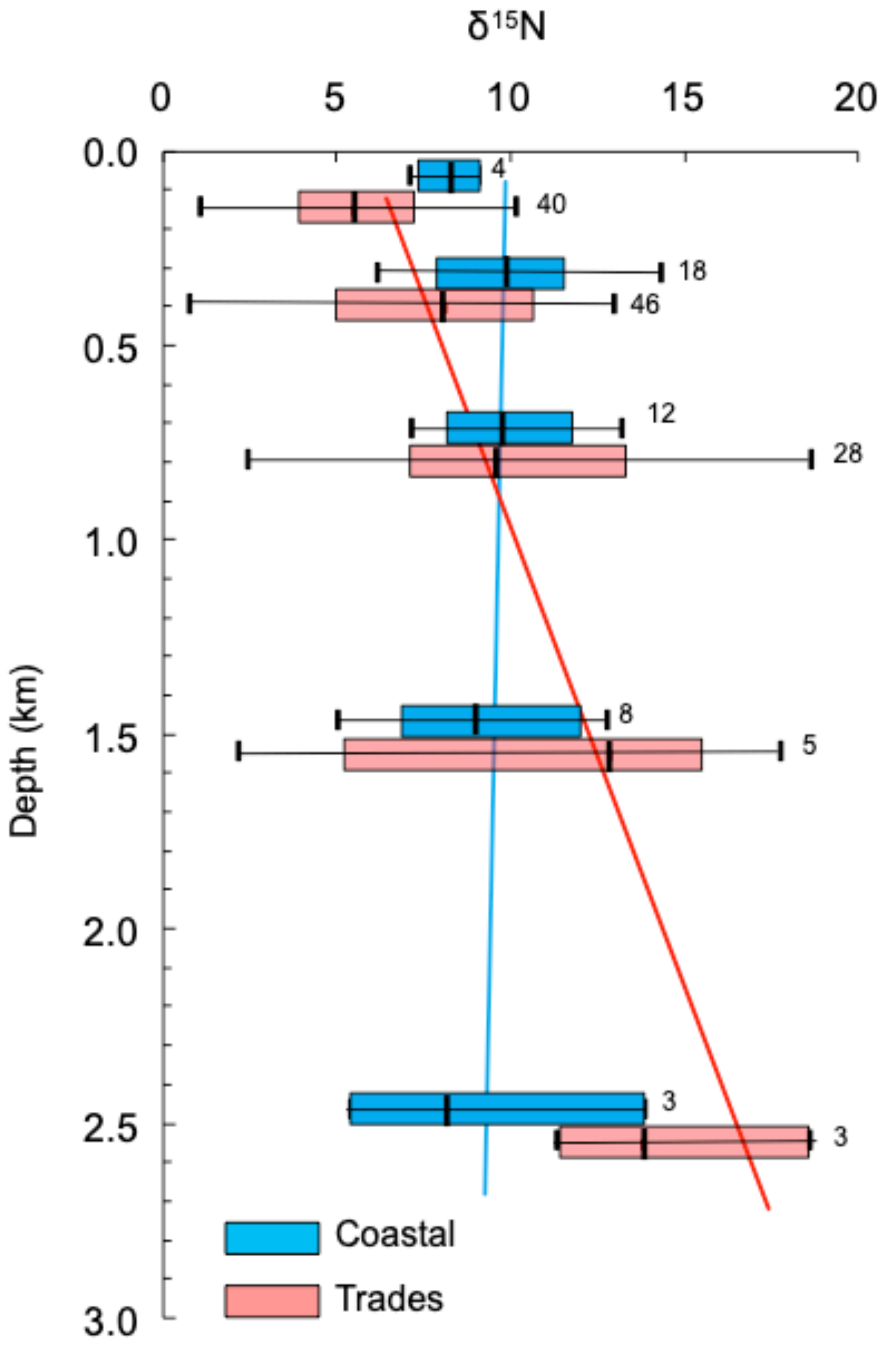

Supplementary Figure 3. Box and whisker vertical profiles of zooplankton $\delta^{15}N$ for Coastal and Trades biomes. For each depth interval, the median is indicated by a thick horizontal line, the box encompasses the 25% and 75% percentiles, and the whiskers the minimum and maximum values. The number of data is indicated for each box. The lines represent the fitted regression equations using all data shown in Supplementary Table 2.

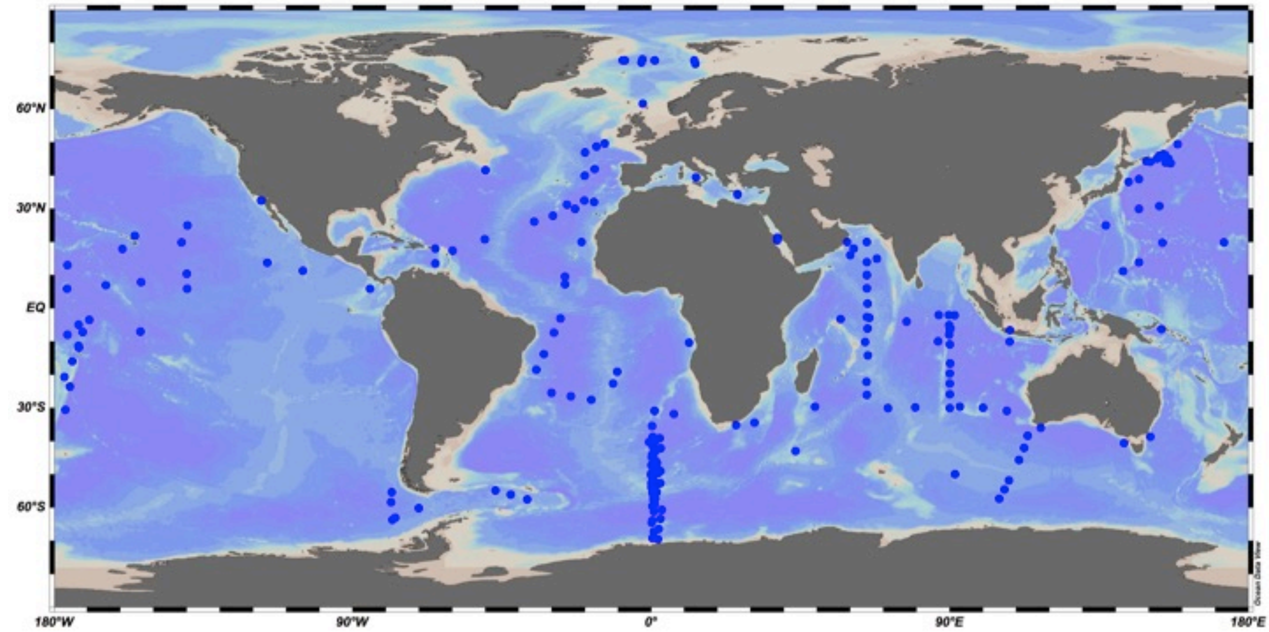

Supplementary Figure 4. Location of stations of reviewed zooplankton vertical profiles in the present study.

References

1. Angel M.V., Baker A. Vertical distribution of the standing crop of plankton and micronekton at three stations in the northeast Atlantic. *Biol. Oceanogr*. **2**, 1-30 (1982).

2. Foxton P. The distribution of the standing crop of zooplankton in the Southern Ocean. *Discovery Rep*. **28**, 191-236 (1956).

3. Vinogradov M.E. Vertical distribution of the Oceanic Zooplankton (Israel Program for Scientific Translations) 333 pp (Jerusalem) (1970)

4. Grice G.D., Hulsemann K. Bathypelagic calanoid copepods of the western Indian Ocean. *Proc. U.S. Natl. Mus*. **122**, 1-67 (1967).

5. Koppelmann R., Schäfer P., Schiebel R. Organic carbon losses measured by heterotrophic activity of mesozooplankton and CaCO 3 flux in the bathypelagic zone of the Arabian Sea. *Deep-Sea Res. II* **47**, 169-187 (2000).

6. Koppelmann R., Weikert H. Full-depth zooplankton profiles over the deep bathyal of the NE Atlantic. *Mar. Ecol. Prog. Ser.* **86**, 263-263 (1992).

7. Koppelmann R., Weikert H. Temporal changes of deep-sea mesozooplankton abundance in the temperate NE Atlantic and estimates of the carbon budget. *Mar. Ecol. Prog. Ser.* **179**, 27-40 (1999).

8. Vinogradov M.E., Shushkina E.A., Nezlin N.P., Arnautov G.N. Vertical distribution of zooplankton in the frontal zone of the Gulf Stream and Labrador Current. *J. Plankton Res*. **20**, 85-103 (1998).

9. Weikert H., Trinkaus S. Vertical mesozooplankton abundance and distribution in the deep Eastern Mediterranean Sea SE of Crete. *J. Plankton Res.* **12**, 601-628 (1990).

10. Weikert H., Koppelmann R., Wiegratz S. Evidence of episodic changes in deep-sea mesozooplankton abundance and composition in the Levantine Sea (Eastern Mediterranean). *J. Mar. Syst.* **30**, 221-239 (2001).

11. Koppelmann R., Weikert H. Spatial and temporal distribution patterns of deep-sea mesozooplankton in the eastern Mediterranean–indications of a climatically induced shift? *Mar. Ecol.* **28**, 259-275 (2007).

12. Koppelmann R., Weikert H., Halsband-Lenk C., Jennerjahn T. Mesozooplankton community respiration and its relation to particle flux in the oligotrophic eastern Mediterranean. *Global Biogeochem. Cycles* **18**, GB1039, doi:10.1029/ 2003GB002121 (2004).

13. Minutoli R., Guglielmo L. Mesozooplankton carbon requirement in the Tyrrhenian Sea: its vertical distribution, diel variability and relation to particle flux. *Mar. Ecol. Prog. Ser.* **446**, 91-105 (2012).

14. Roe H.S.J. Midwater biomass profiles over the Madeira Abyssal Plain and the contribution of copepods. *Hydrobiologia* **167**, 169-181 (1988).

15. Grice G.D., Hulsemann K. Abundance, vertical distribution and taxonomy of calanoid copepods at selected stations in the northeast Atlantic. *Proc. Zool. Soc. London* **146**, 213-262 (1965).

16. Yamaguchi A., Watanabe Y., Ishida H., Harimoto T., Furusawa K., Suzuki S., Takahashi M. Latitudinal differences in the planktonic biomass and community structure down to the greater depths in the western North Pacific. *J. Oceanography* **60**, 773-787 (2004).

17. Wishner K.F. The biomass of the deep-sea benthopelagic plankton. *Deep-Sea Res.* **27**, 203-216 (1980).

18. Weikert H. The Vertical Distribution of Zooplankton in Relation to Habitat Zones in the Area of the Atlantis II Deep, Central Red Sea. *Mar. Ecol. Prog. Ser.* **8**, 129-143 (1982).

19. Böttger-Schnack R. Vertical structure of small metazoan plankton, especially non-calanoid copepods. I. Deep Arabian Sea. *J. Plankton Res*. **18**, 1073-1101 (1996).

20. Richter C. Regional and seasonal variability in the vertical distribution of mesozooplankton in the Greenland Sea. *Ber. Polarforsch.* **154**, 1-90 (1994).

21. Martin, J.H., Knauer, G.A., Karl, D.M., Broenkow, W.W. VERTEX: Carbon cycling in the northeast Pacific. *Deep-Sea Res.* **34**, 267-285 (1987).

22. Knauer, G.A., Redalje, D.G., Harrison, W.G., Karl, D.M. New production at the VERTEX time-series site. *Deep-Sea Res.* **37**, 1121-1134 (1990).

23. Karl, D.M., Christian, J.R., Dore, J.E., Hebel, D.V., Letelier, R.M., Tupas, L.M., Winn, C.D. Seasonal and interannual variability in primary production and particle flux at Station ALOHA. *Deep-Sea Res. II* **43**, 539-568 (1996).

24. Hernández-León, S., Olivar, P., Fernández de Puelles, M.L., Bode, A., Castellón, A., López-Pérez, C., Tuset, V.M., González-Gordillo, J.I. Zooplankton and micronekton active flux across the tropical and subtropical Atlantic Ocean. *Front. Mar. Sci.* **6**, 535, doi: 10.3389/ fmars.2019.00535 (2019).

25. Miquel, J.C., Martín, J., Gasser, B., Rodriguez-y-Baena, A., Toubal, T., Fowler, S.W. Dynamics of particle flux and carbon export in the northwestern Mediterranean Sea: A two decade time-series study at the DYFAMED site. *Progress oceanogr* **91**, 461-481 (2011).

26. Buesseler, K.O., Lamborg, C.H., Boyd, P.W., Lam, P.J., Trull, T.W., Bidigare, R.R., Bishop, J.K.B., Casciotti, K.L., Dehairs, F., Elskens, M., Honda, M., Karl, D.M., Siegel, D.A., Silver, M.W., Steinberg, D.K., Valdes, J., Van Mooy, B., Wilson, S. Revisiting carbon flux through the ocean's twilight zone. *Science*, **316**(5824), 567-570 (2007).

27. Guidi L., Legendre L., Reygondeau G., Uitz J., Stemmann L., Henson S.A. A new look at ocean carbon remineralization for estimating deep-water sequestration. *Global Biogeochem. Cycles* **29**, 1044-1059 (2015).

28. Henson S.A., Sanders R., Madsen E. Global patterns in efficiency of particulate organic carbon export and transfer to the deep ocean. *Global Biogeochem. Cycles* **26**, GB1028, doi:10.1029/2011GB004099 (2012).

29. Lutz M.J., Caldeira K., Dunbar R.B., Behrenfeld M.J. Seasonal rhythms of net primary production and particulate organic carbon flux to depth describe the efficiency of biological pump in the global ocean. *J. Geophys. Res.* **112**, C10011, doi:10.1029/2006JC003706 (2007).

30. Honjo S., Manganini S.J., Krishfield R.A., Francois R. Particulate organic carbon fluxes to the ocean interior and factors controlling the biological pump: A synthesis of global sediment trap programs since 1983. *Progr. Oceanogr*. **76**, 217-285 (2008).

31. Hidaka K., Kawaguchi K., Murakami M., Takahashi M. Downward transport of organic carbon by diel migratory micronekton in the western equatorial Pacific: its quantitative and qualitative importance. *Deep-Sea Res. I* **48**, 1923-1939 (2001).

32. Ariza A.V., Landeira J.M., Escánez A., Wienerroither R.M., Aguilar N., Røstad A., Kaartvedt S., Hernández-León S. Vertical distribution, composition and migratory patterns of acoustic scattering layers in the Canary Islands. *J. Mar. Syst.* **157**, 82-91 (2016).

33. Nagata, T., Fukuda, H., Fukuda, R., Koike, I. Bacterioplankton distribution and production in deep Pacific waters: large–scale geographic variations and possible coupling with sinking particle fluxes. *Limnol. Oceanogr.* **45**, 426-435 (2000).

34. Santana-Falcón, Y., Álvarez-Salgado, X.A., Pérez-Hernández, M.D., Hernández-Guerra, A., Mason, E., Arístegui, J. Organic carbon budget for the eastern boundary of the North Atlantic subtropical gyre: major role of DOC in mesopelagic respiration. *Sci. rep.* **7**, *10129* DOI:10.1038/s41598-017-10974-y (2017).

35. Arístegui J., Agustí S., Duarte C.M. Respiration in the dark ocean. *Geophys. Res. Lett.* **30**, 1041, doi:10.1029/2002GL016227 (2003a).

36. Arístegui, J., Barton, E.D., Montero, M.F., García-Muñoz, M., Escánez, J. Organic carbon distribution and water column respiration in the NW Africa-Canaries Coastal Transition Zone. *Aquat. Microbial Ecol.* **33**, 289-301 (2003b).

37. Jenkins, W.J., Wallace, D.W.R. Tracer based inferences of new primary production in the sea. In *Primary productivity and biogeochemical cycles in the sea* (pp. 299-316). Springer, Boston, MA (1992).

38. Packard, T.T., Denis, M., Rodier, M., Garfield, P. Deep-ocean metabolic CO2 production: calculations from ETS activity. *Deep-Sea Res.* **35**, 371-382 (1988).

39. Savenkoff, C., Prieur, L., Reys, J.P., Lefevre, D., Dallot, S., Denis, M. Deep microbial communities evidenced in the Liguro-Provençal front by their ETS activity. *Deep-Sea Res. I* **40**, 709-725 (1993).

40. Lefevre, D., Denis, M., Lambert, C.E., Miquel, J.C. Is DOC the main source of organic matter remineralization in the ocean water column?. *J. Mar. Syst.* **7**, 281-291 (1996).

41. Baltar, F., Arístegui, J., Gasol, J.M., Sintes, E., Herndl, G.J. Evidence of prokaryotic metabolism on suspended particulate organic matter in the dark waters of the subtropical North Atlantic. *Limnol. Oceanogr.* **54**, 182-193 (2009).

42. Fernández-Castro, B., Arístegui, J., Anderson, L., Montero, M.F., Hernández-León, S., Marañón, E., Mourino-Carballido, B. Mesopelagic respiration near the ESTOC (European Station for Time-Series in the Ocean, 15.5^{o} W, 29.1^{o} N) site inferred from a tracer conservation model. *Deep-Sea Res. I* **115**, 63-73 (2016).

43. Hernández-León, S., Ikeda, T. A global assessment of mesozooplankton respiration in the ocean. *J. Plankton Res.* **27**, 153-158 (2005).

44. Childress, J.J., Thuesen, E.V. Metabolic potential of deep-sea animals: regional and global scales. In *Deep-sea food chains and the global carbon cycle* (pp. 217-236). Springer, Dordrecht (1992).

45. Helmke, P., Romero, O., Fischer, G. Northwest African upwelling and its effect on offshore organic carbon export to the deep sea. *Global Biogeochem. Cycles* **19,** GB4015, doi:10.1029/2004GB002265 (2005).

46. Packard, T.T., Osma, N., Fernández-Urruzola, I., Codispoti, L.A., Christensen, J.P., Gómez, M. Peruvian upwelling plankton respiration: calculations of carbon flux, nutrient retention efficiency, and heterotrophic energy production. *Biogeosciences* **12**, 2641–2654 (2015)

47. Turley, C.M., Mackie, P.J. Biogeochemical significance of attached and free-living bacteria and the flux of particles in the NE Atlantic Ocean. *Mar. Ecol. Progr. Ser.* **115**, 191-191 (1994).
