# Supplementary material for: Large deep-sea zooplankton biomass mirrors primary production in the global ocean
Source: Nat Commun. 2020 Nov 27;11:6048. doi: 10.1038/s41467-020-19875-7 (PMC7695708; doi:10.1038/s41467-020-19875-7)
Supplement: Supplementary file 3 — Reporting Summary [file 41467_2020_19875_MOESM3_ESM.pdf]

nature research

Corresponding author(s): Santiago Hernández-León

Last updated by author(s): Oct 15, 2020

Reporting Summary

Nature Research wishes to improve the reproducibility of the work that we publish. This form provides structure for consistency and transparency in reporting. For further information on Nature Research policies, see Authors & Referees and the Editorial Policy Checklist .

Statistics

For all statistical analyses, confirm that the following items are present in the figure legend, table legend, main text, or Methods section.

n/a	Confirmed	
☐	☒	The exact sample size (*n*) for each experimental group/condition, given as a discrete number and unit of measurement
☐	☒	A statement on whether measurements were taken from distinct samples or whether the same sample was measured repeatedly
☐	☒	The statistical test(s) used AND whether they are one- or two-sided<br>*Only common tests should be described solely by name; describe more complex techniques in the Methods section.*
☒	☐	A description of all covariates tested
☐	☒	A description of any assumptions or corrections, such as tests of normality and adjustment for multiple comparisons
☐	☒	A full description of the statistical parameters including central tendency (e.g. means) or other basic estimates (e.g. regression coefficient) AND variation (e.g. standard deviation) or associated estimates of uncertainty (e.g. confidence intervals)
☐	☒	For null hypothesis testing, the test statistic (e.g. *F*, *t*, *r*) with confidence intervals, effect sizes, degrees of freedom and *P* value noted<br>*Give P values as exact values whenever suitable.*
☒	☐	For Bayesian analysis, information on the choice of priors and Markov chain Monte Carlo settings
☒	☐	For hierarchical and complex designs, identification of the appropriate level for tests and full reporting of outcomes
☒	☐	Estimates of effect sizes (e.g. Cohen's *d*, Pearson's *r*), indicating how they were calculated

Our web collection on statistics for biologists contains articles on many of the points above.

Software and code

Policy information about availability of computer code

Data collection	Some data were obtained from original figures using GraphClick software v3.0 (see text)
Data analysis	Statistica v7.0 and R package (2013) were used for analysis and statistics, and ZooImage1 v1.2-1 to measure abundance of zooplankton. LADCP data were processed with IFM-GEOMAR LADCP/LDEO V10 software.

For manuscripts utilizing custom algorithms or software that are central to the research but not yet described in published literature, software must be made available to editors/reviewers. We strongly encourage code deposition in a community repository (e.g. GitHub). See the Nature Research guidelines for submitting code & software for further information.

Data

Policy information about availability of data

All manuscripts must include a data availability statement. This statement should provide the following information, where applicable:

- Accession codes, unique identifiers, or web links for publicly available datasets
- A list of figures that have associated raw data
- A description of any restrictions on data availability

Data was submitted to PANGEA repository:
Hernández-León, Santiago; Bode, Antonio; Irigoien, Xabier; Echevarría, Fidel; Fernández de Puelles, Maria Luz; González-Gordillo, Ignacio; Acuña, José Luis (2020). Zooplankton biomass during the Malaspina Cruise. PANGAEA, https://doi.org/10.1594/PANGAEA.922974

Hernández-León, Santiago; Koppelmann, Rolf (2020). Review of global zooplankton biomass in the bathypelagic zone. PANGAEA, https://doi.org/10.1594/PANGAEA.923149

Hernández-León, Santiago (2020). Carbon export and sequestration in the ocean. PANGAEA, https://doi.org/10.1594/PANGAEA.923832

Fraile-Nuez, Eugenio; Hernández-León, Santiago (2020). Lowered Acoustic Doppler Current Profiler (LADCP) backscatter attached to the rosette sampler during the Malaspina Circumnavigation Expedition. Spanish Institute of Oceanography, PANGAEA, https://doi.org/10.1594/PANGAEA.922619

Bode, Antonio; Mompeán, Carmen (2020). Stable isotope data of mesozooplankton for depth layers along the Malaspina-2010 expedition. PANGAEA, https://doi.org/10.1594/PANGAEA.919314

Field-specific reporting

Please select the one below that is the best fit for your research. If you are not sure, read the appropriate sections before making your selection.

☐ Life sciences ☐ Behavioural & social sciences ☒ Ecological, evolutionary & environmental sciences

For a reference copy of the document with all sections, see nature.com/documents/nr-reporting-summary-flat.pdf

Ecological, evolutionary & environmental sciences study design

All studies must disclose on these points even when the disclosure is negative.

Study description	We reviewed 274 profiles of zooplankton biomass smaller than 5 mm when this information was available but always excluding macrozooplankton and micronekton. Natural abundance of stable nitrogen isotopes was expressed as δ15N (‰) relative to atmospheric nitrogen, and determined in zooplankton specimens collected from subsamples of the different water layers. Specific respiration rates were derived from the measurement of the electron transfer system (ETS) activity in those samples from the Malaspina cruise with enough biomass at bathypelagic depths to obtain a subsample and perform the enzymatic assay. Backscatter intensity was measured using a Lowered Acoustic Doppler Current Profiler (LADCP) system equipped with two 300 kHz Teledyne/RDI Workhorses, which were mounted on a CTD rosette sampler and deployed at each cast. Surface integrated primary production was downloaded from the Ocean Productivity web site (http://www.science.oregonstate.edu/ ocean.productivity/index.php) using the Vertical Generalized Production Model (VGPM).
Research sample	Zooplankton biomass, stable isotopes, enzymatic activities, LADCP acoustics, and primary production (see Methods). A fraction of the zooplankton sample was used for analysis of biomass and enzymatic activity. Zooplankton specimens for stable isotope analysis were selected from samples collected in different depth layers between the surface and 2500 m covering a large range of trophic positions: from herbivores to secondary carnivores. Primary production was obtained from available remote sensing data (see text) and acoustics using the IFM-GEOMAR LADCP/LDEO V10 software given above.
Sampling strategy	Global distribution of zooplankton biomass at different depth layers. Zooplankton net samples normally produce a large sample as they filter an important amount of ocean water, and therefore, sample size is considered quite large for our purposes. In deep waters the zooplankton abundance and biomass is lower but when it was small it was only used for abundance and taxonomy and no for isotopes or enzymatic analysis. Here, we only present data when enough sample was obtained for each parameter.
Data collection	Literature review and data collected along the Malaspina cruise around the globe.
Timing and spatial scale	Global distribution of zooplankton biomass at epi- (0-200 m layer), meso- (200-1000 m layer), and bathypelagic (>1,000 m deep) depths based on data acquired during the Malaspina Circumnavigation Expedition, which surveyed zooplankton biomass in subtropical and tropical oceans during 2010 and 2011, amended with published estimates of deep-sea zooplankton biomass (see Methods). The Malaspina Circumnavigation Expedition started in December 2010 and finished in July 2011, and sampled in tropical and subtropical waters.
Data exclusions	No data was excluded from the analysis.
Reproducibility	Not applicable as this is a field sampling as usual in oceanography.
Randomization	Not applicable as it is a field sampling in oceanography.
Blinding	Not applicable as it is a field sampling in oceanography.

Did the study involve field work? ☒ Yes ☐ No

Field work, collection and transport

Field conditions	Global distribution of zooplankton biomass at different depth layers surveying zooplankton biomass in subtropical and tropical oceans during 2010 and 2011, amended with published estimates of deep-sea zooplankton biomass (see Methods).
Location	Tropical and subtropical Atlantic, Pacific, and Indian oceans.
Access and import/export	No ethical approval was required as we sampled invertebrates.
Disturbance	Not applicable as we did not disturb the ocean while sampling.

Reporting for specific materials, systems and methods

We require information from authors about some types of materials, experimental systems and methods used in many studies. Here, indicate whether each material, system or method listed is relevant to your study. If you are not sure if a list item applies to your research, read the appropriate section before selecting a response.

Materials & experimental systems

n/a	Involved in the study
☒	☐ Antibodies
☒	☐ Eukaryotic cell lines
☒	☐ Palaeontology
☐	☒ Animals and other organisms
☒	☐ Human research participants
☒	☐ Clinical data

Methods

n/a	Involved in the study
☒	☐ ChIP-seq
☒	☐ Flow cytometry
☒	☐ MRI-based neuroimaging

Animals and other organisms

Policy information about studies involving animals; ARRIVE guidelines recommended for reporting animal research

Laboratory animals	Not applicable as no laboratory animals were used in the study.
Wild animals	Organisms captured during the Malaspina circumnavigation were collected using a 0.5 m2 Multinet Sampler (Hydrobios) equipped with 5 nets of 300 µm mesh and a flowmeter to measure the volume of water filtered. Stratified tows were performed by day in vertical hauls from 3000 m to the surface. Samples were then fixed on board and stored in a 4% buffered formalin and seawater for further analysis. Samples for enzymatic activities were frozen inmediately in liquid nitrogen.
Field-collected samples	No living animals were used for experimentation.
Ethics oversight	No ethical approval was required as we sampled invertebrates.

Note that full information on the approval of the study protocol must also be provided in the manuscript.
